# Roles of Membrane Domains in Integrin-Mediated Cell Adhesion

**DOI:** 10.3390/ijms21155531

**Published:** 2020-08-01

**Authors:** Daniel Lietha, Tina Izard

**Affiliations:** 1 Cell Signaling and Adhesion Group, Structural and Chemical Biology, Margarita Salas Center for Biological Research (CIB-CSIC), E-28040 Madrid, Spain; daniel.lietha@cib.csic.es; 2 Cell Adhesion Laboratory, Department of Integrative Structural and Computational Biology, The Scripps Research Institute, Jupiter, FL 33458, USA

**Keywords:** adhesion, integrin signaling, liquid-order, liquid-disorder, lipid rafts

## Abstract

The composition and organization of the plasma membrane play important functional and regulatory roles in integrin signaling, which direct many physiological and pathological processes, such as development, wound healing, immunity, thrombosis, and cancer metastasis. Membranes are comprised of regions that are thick or thin owing to spontaneous partitioning of long-chain saturated lipids from short-chain polyunsaturated lipids into domains defined as ordered and liquid-disorder domains, respectively. Liquid-ordered domains are typically 100 nm in diameter and sometimes referred to as lipid rafts. We posit that integrin β senses membrane thickness and that mechanical force on the membrane regulates integrin activation through membrane thinning. This review examines what we know about the nature and mechanism of the interaction of integrins with the plasma membrane and its effects on regulating integrins and its binding partners.

## 1. Introduction

Biomembranes are a key component of life, as they allow the formation of compartments to provide conditions required for biochemical reactions, and also provide an interaction platform for a multitude of key cellular processes. The membranes consist of phospholipids, glycolipids, sterols, and proteins, arranged in a lipid bilayer. The bilayer architecture is a consequence of the amphipathic character of its constituents, which in an aqueous environment with polar regions oriented towards the aqueous phase and hydrophobic regions facing each other. 

The concept of the bilayer was first postulated in 1935 [1] and then replaced with the fluid mosaic model in 1972 [2]. The latter model is in large part still accepted and describes biomembranes as fluid objects where lipids and proteins are in motion and can freely diffuse along the plane of the lipid bilayer. One main adjustment to the fluid mosaic model of biomembranes is the fact that they have a very high protein content [3,4]. This molecular crowding is increasingly described to occur also in soluble states [5] and drives the separation of phases of distinct contents. The non-homogenous distribution of proteins within the membrane is amplified further through interactions on either side of the membrane. 

Islands with distinct compositions compared to the rest of the fluid mosaic biomembrane are considered membrane microdomains. Lipid rafts, wherein the spontaneous partitioning of long-chain saturated lipids from short-chain polyunsaturated lipids results in thicker liquid-ordered and thinner liquid-disordered domains, respectively [6,7], as noted by Hansen [8], are the best-characterized membrane microdomain. The fact that lipid rafts do not solubilize in nonionic detergents like triton X-100 at 4 °C and are thus called detergent-resistant membranes [9,10,11] makes them especially difficult to study. 

**Figure 1:**
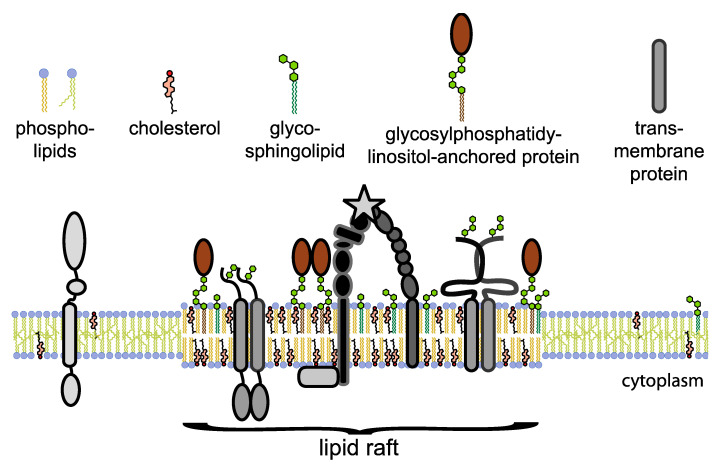
Schematic of lipid raft organization. The asymmetric plasma membrane contains phospholipids, glycosphingolipids, cholesterol, and protein receptors that are organized in the thicker lipid microdomains. These lipid rafts compartmentalize cellular processes and signal transduction by organizing and concentrating signaling molecules to more favorably interact with protein receptors as well as effectors. Lipid rafts float freely in the plasma membrane while being packed tighter and more ordered compared to non-raft regions.

Lipid rafts are rich in cholesterol and sphingolipids as well as in a variety of proteins [12,13], including lipid-linked proteins such as glycosylphosphatidylinositol-anchored proteins and signaling molecules. Thus, they provide an essential platform for cell signaling processes [10] and play important roles in the regulation of cell adhesion to the extracellular matrix and associated cell migration by providing a scaffold that concentrates adaptor and scaffolding proteins, as well as the actin cytoskeleton, effectors, kinases, and receptors to trigger cancer signaling events [14] (Figure 1). By concentrating signaling molecules such as the src family of non-receptor tyrosine kinases as well as the small GTPase rac1, lipid raft microdomains regulate the extracellular matrix-mediated cell migration and direct signaling pathways of cell division, cell shape, cell motility, and cell adhesion [10,13,15,16,17,18,19,20,21,22,23,24,25]. Specifically, lipid rafts organize signaling molecules and provide platforms for cell adhesion signaling. Several studies show that integrins are associated with lipid rafts [26,27,28] and this interaction is important for triggering signaling cascades upon cell attachment to the extracellular matrix.

Cell adhesion to the extracellular matrix is mediated by the integrin transmembrane receptor, a heterodimer composed of an α and a β subunit. Integrins attach to extracellular matrix components, such as collagen or fibronectin, and assemble a large focal adhesion protein complex that links the adhesion sites to the actin cytoskeleton. When bound to the extracellular matrix, integrins transmit signals within the cell that control cell spreading, retraction, migration, and proliferation. These signals drive many physiological and pathological processes, such as development, wound healing, immunity, thrombosis, and cancer metastasis. 

In this review we discuss the role of lipid rafts in integrin-mediated cell adhesion and how lipid rafts can affect integrin structure and signaling. Although the importance of lipid rafts in cell adhesion has long been recognized, the lack of mechanistic insights has prevented a clear view of how lipid rafts are linked to integrin function. Here we focus on recent findings that have helped to postulate a detailed mechanistic model that can explain how lipid rafts selectively include activated integrin receptors and hence provide a platform for active integrin signaling.

## 2. Cytoskeletal Rearrangements

**Figure 2:**
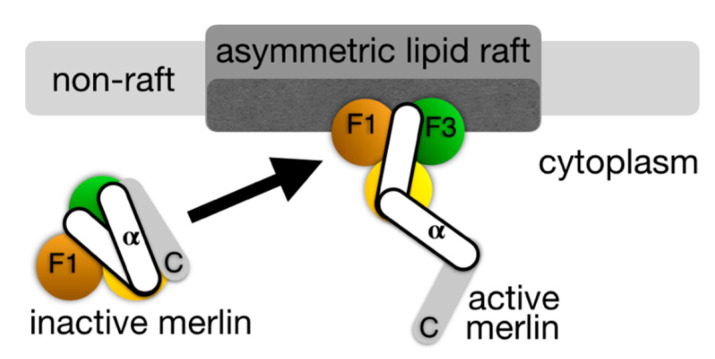
Proposed merlin activation mechanism in lipid rafts. Merlin belongs to the ezrin-radixin-moesin (ERM) family of proteins that are characterized by their *N*-terminal four point one, ezrin, radixin, moesin (FERM) head domain, a central α-helical domain and a *C*-terminal tail domain that binds to the actin cytoskeleton. The FERM domain is comprised of three subdomains, F1, F2, and F3 that are arranged in a cloverleaf-like structure (depicted spectrally; F1, orange; F2, yellow; F3, green). The *C*-terminal domain of merlin differs from other ERM proteins and does not contain an F-actin binding domain. **Left***,* the tumor suppressor protein merlin is inactive in its closed conformer. **Right***,* upon binding to the PI(4,5)P_2_ that is found in lipid rafts, merlin is activated by severing its head-tail interaction, thereby resulting in its open conformation and tumor suppressor functions [36].

The F-actin cytoskeleton interacts and controls many structural and functional aspects of the membrane lipid rafts. For example, integrin α5 translocation and activation were prevented by the disruption of the F-actin-based cytoskeleton and knockdown of caveolin-1. Lipid rafts play a role in many cellular processes including cell proliferation [29,30,31] and act as a sorting platform for proteins, especially those involved in cancer (ovarian, prostate, and renal cell carcinoma). Their association with the actin cytoskeleton drives tumor progression. Thus, molecules that interfere with the assembly of the actin cytoskeleton with lipid rafts might have anticancer activities. In general, the cytoskeletal rearrangement that stabilizes lipid rafts and the recruitment of cytoskeletal proteins to the ordered microdomains facilitates increased intermolecular interactions that are crucial during the development of cancer. Since cholesterol and saturated sphingolipids increase the rigidity of lipid rafts, their protein compartmentalization might stabilize interactions of raft components [32]. Tumor suppressors that regulate lipid rafts might reduce the attachment of the cytoskeleton to the membrane [33]. One such tumor suppressor, the protein that is responsible for neurofibromatosis 2, termed merlin (or schwannomin or neurofibromin 2), disrupts such membrane interactions with the actin cytoskeleton [34,35]. The inactive closed merlin conformation is found to be associated with non-raft regions of the plasma membrane, while merlin activation by severing the merlin head-tail intramolecular interaction seems to be associated with lipid rafts [36] (Figure 2).

## 3. Integrin Structure

**Figure 3:**
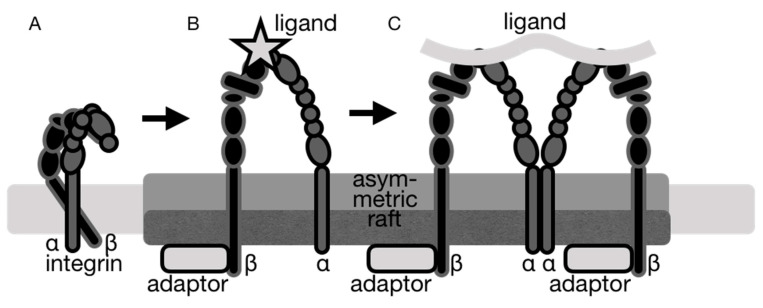
Integrin adhesion and signaling are linked to the recruitment of integrins to lipid rafts. Several integrins have been found in lipid rafts, whereby the activated form of integrin preferably localizes to cholesterol-enriched lipid rafts. The cholesterol-rich membrane domains cluster integrin at focal adhesions, which regulates integrin activation. (**A**)*,* inactive integrin localized to non-raft regions of the plasma membrane. (**B**), activated integrin localizes to lipid rafts. (**C**), integrin clustering at focal adhesions in lipid rafts.

Integrins are single-pass αβ heterodimeric transmembrane receptors that are composed of a large extracellular ligand-binding domain and short cytoplasmic tail domains (Figure 3A) [37]. Their single transmembrane α-helices engage in intermolecular interactions when integrin is in its resting state. Upon activation with extracellular ligands (Figure 3B,C), integrins change from their low-affinity binding to their high-affinity binding state, which modifies cell adhesion [38]. 

Structural studies on many integrins showed that this integrin exists in a continuous conformational equilibrium ranging from a compact conformation to a fully extended conformer with the cytoplasmic tail domains separated upon binding to extracellular ligands [39,40,41,42,43,44,45,46,47,48,49,50,51,52,53,54,55,56,57,58,59,60]. High-resolution structural data are perhaps more available for the inactive integrin conformer and for the transmembrane domains, where we know that the longer transmembrane α-helix of the β subunit is tilted relative to the shorter α-helix of the α subunit that it binds to [61] (Figure 3A). In the active integrin conformation, it is thought that the angle of the transmembrane α-helix of the β subunit is distinct from that of its tilt seen in the inactive when bound to the α subunit [62]. Hinging movements of the integrin transmembrane domains during integrin activation were speculated over two decades ago [40,41,44,63,64]. For example, movements of the membrane proximal region in and out of the membrane were suggested to provide a venue for integrin signaling [65]. The nuclear magnetic resonance (NMR) structure of the transmembrane domain of integrin β3 in phospholipid bicelles and detergent micelles revealed that it forms a 30-residue α-helix that is embedded in the hydrophobic bicelle core. The length of this transmembrane α-helix suggested a pronounced tilt within a typical lipid bilayer, whereby the charged lysine (residue 716) snorkels out of the lipid core while hydrophobic residues (residues 717 through 721), in particular leucines, remain immersed in the membrane. This NMR structure of the transmembrane domains of the non-covalently-associated integrin αIIbβ3 in small bicelles also revealed that a so-called inner membrane clasp (IMC) stabilizes the integrin heterodimer at the intracellular side, while this role is performed by the ectodomain and outer membrane clasp (OMC) on the extracellular side [66]. The structure suggested a straight α-helix for integrin α and an α-helix tilt of ~25° for integrin β. The α-helix tilt of integrin β might control bidirectional transmembrane signaling and changes in the thickness of the membrane seem to affect integrin signaling by modulating the tilt angle. 

We posit that thicker membranes might force integrin repartitioning. The membrane bilayer clearly plays a role in the integrin activation process [67]. For example, the binding of cholesterol to glycosylphosphatidylinositol-anchored proteins and lymphocyte function-associated antigen 1 (LFA-1) integrin (also known as integrin αLβ2 or CD11a/CD18) was visualized by single-molecule near-field optical microscopy in immune cells [68]. Additionally, upon binding to lipid raft components, the integrin conformation is altered [37,69,70] and thus function. For example, active integrin α4 of integrin α4β1 bound cholesterol to then mediate adhesion of T lymphocytes [71]. 

## 4. Integrin Signaling

Integrins are bidirectional transmembrane signaling and adhesion metalloprotein receptors involved in cell survival, cell migration, cell proliferation, and cell differentiation [72,73]. Integrins play key roles in hematopoiesis, vascular development, immune and inflammatory responses, as well as hemostasis and arterial thrombosis. By binding to the extracellular matrix, integrins regulate cellular responses to several physical and chemical cues [74] that control a variety of biological processes. The binding of integrins to extracellular ligands is stabilized by the binding to intracellular scaffolding proteins such as vinculin or talin, thereby linking integrins to the actin cytoskeleton and mediating mechano-transduction [75,76]. 

**Figure 4:**
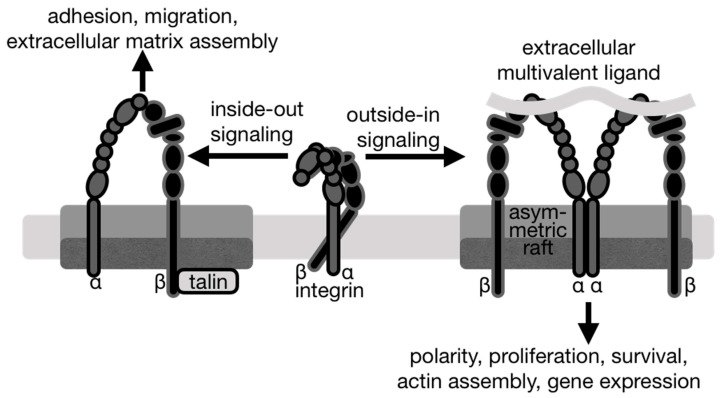
Integrins signal in both directions with different outcomes. **Left,** inside-out integrin signaling is initiated by the binding of an intracellular activator such as talin or kindlin to the integrin β cytoplasmic tail domain. This leads to conformational changes that activate integrins by increasing the integrin affinity for extracellular ligands. The resulting strong interactions between integrins and the extracellular matrix enable integrins to transmit forces for extracellular matrix remodeling and assembly as well as cell migration. **Center**, in its inactive, low-affinity state, the integrin is in a bent conformation. **Right,** during traditional outside-in signaling, multivalent extracellular ligands bind to integrins, thereby causing a conformational change and integrin clustering.

Integrins signal in both directions with distinct biological outcomes (Figure 4). Inside-out integrin signaling is initiated by the binding of an intracellular activator. For example, the binding of talin or kindlins to the integrin β cytoplasmic tail domain leads to conformational changes that activate integrins by increasing the integrin affinity for extracellular ligands. The resulting strong interactions between integrins and the extracellular matrix enable integrins to transmit forces for extracellular matrix remodeling and assembly as well as cell migration. 

In contrast, during traditional outside-in signaling, extracellular ligands bind to integrins, causing a conformational change and integrin clustering due to the multivalent nature of the ligands. Both inside-out and outside-in signaling lead to the regulation of cell polarity, cell survival, and cell proliferation, as well as gene expression and the remodeling of the actin cytoskeleton [77]. There are several proteins that interact with integrins within lipid rafts and an important question remains, how mechanistically lipid rafts affect these processes.

## 5. Lipid Rafts in Integrin Partitioning

Lipid rafts are involved in integrin-mediated signal transduction pathways initiated by cell adhesion [16,78,79], as well as in integrin clustering at focal adhesions, which regulates integrin activation (38). Lipid rafts regulate integrin signaling by partitioning activated integrins in microdomains where they form specific interactions with upstream and downstream signaling molecules [80,81,82]. The recruitment of integrins α4β1 and αLβ2 to lipid raft domains occurs specifically during inside-out signaling, upon binding to and activation by extracellular matrix ligands [71]. These integrins are excluded from lipid rafts without stimulation but mobilized to the lipid raft compartment upon stimulation [71]. In oligodendrocytes, activation of integrin α6β1 with manganese increases integrin α6β1 concentration in lipid rafts [83]. Lipid rafts might sequester activated integrins by providing a more favorable membrane environment for the distinct conformation of activated integrins since it is the activated form of integrin that preferentially localizes to the cholesterol-enriched membrane lipid rafts [83] (Figure 3 and Figure 4). While little is known about the interaction of the integrin transmembrane α-helices with membrane lipids, an invariant lysine at the *C*-terminus of both integrin subunits seems to be important for the lateral mobility of integrins in the membrane [65]. 

Several integrins have been found in lipid rafts, such as integrins LFA-1 [26,71], αVβ3 [27], α6β4 [84], and β1 [85,86], also in α6β1 [87]. Platelet integrin αIIbβ3 partitions in the 1,2-dioleoyl-*sn*-glycero-3-phosphocholine-rich liquid-disordered phase and is excluded from the cholesterol- and sphingomyelin-rich liquid-ordered phase [88]. Interestingly, activated platelets form lipid rafts that act as foci and integrate adhesion and signaling molecules [89]. In leukocytes, integrin activation leads to lipid raft association, which again has a role in cell adhesion [71]. Further, integrin α5 translocates into lipid rafts upon activation [90]. In rat fibroblasts, changes of phospholipids, cholesterol levels, and membrane fluidity reduced binding of integrin α5β1 to fibronectin at focal adhesions [91]. In T cells, integrins α4β1 and αLβ2 colocalize with the ganglioside lipid raft marker [71]. Cell surface integrins have also been shown to localize to lipid rafts [16,26,27,71]. Integrins α6β4 and αLβ2 are enriched in lipid rafts, whereby integrin α6β4 localized in rafts promotes movement of integrin α6β4 to the rafts [92].

Loss of cell adhesion alters the localization and raft partitioning of many non-raft molecules [93], including integrins [16,26,27,71]. In migrating cells, lipid rafts preferentially localize at the leading edges where new integrin-mediated adhesion to the extracellular matrix sites is occurring [94]. For example, during tumor cell migration of melanoma cells, lipid rafts control lamellipodia formation through the cytoskeleton-mediated recruitment of integrins β1 and β3 to the leading edge [95]. Additionally, lipid rafts might play a crucial role in platelet-derived growth factor receptor α-mediated regulation of differentiation based on integrin partitioning. For example, in oligodendrocytes, the platelet-derived growth factor receptor α binds to integrin αVβ3, thereby promoting proliferation. Upon differentiation, most of platelet-derived growth factor receptor α localizes to rafts, where it binds to integrin α6β1, thereby switching to survival [87]. Further, integrin-mediated adhesion signaling appears to be involved in various causes of neural injury [96]. Integrin αVβ3 is upregulated in astrocytes upon neural injury and inflammation, leading to astrocyte activation and migration via αVβ3 integrin interactions with the receptor thymocyte differentiation antigen 1 (Thy-1)/cluster of differentiation 90 (CD90) in neurons [97]. In this process, αVβ3 integrin restricts Thy-1/CD90 into nanoclusters, which activates neural signaling *via* RhoA and Rho-associated protein kinase (ROCK) to induce altered actin dynamics and neurite retraction [98].

## 6. Integrins and Lipid Raft Components

Integrin-mediated cell adhesion requires intact membrane domains and is dependent on the lipid raft components such as cholesterol, phospholipids, sphingolipids, glycosylphosphatidylinositol-anchored proteins, and the actin cytoskeleton [18,99], as well as divalent cations such as calcium and manganese [100]. Despite decades of studies on integrin activation, the roles that the lipids play in the plasma membrane, in integrin inside-out and outside-in activation steps, have not been fully studied, probably due to the difficulty of working with detergent-resistance membranes. Biochemical experiments might be aided by the use of lipid compositions that more closely resemble the platelet cell membrane by having biomimetic lipid compositions of the platelet membrane [101,102]. In recent years, some insights into the interactions between integrins and lipid raft components have been described. The first lipid ligand for integrin, oxysterol 25-hydrocholesterol, was recently shown to bind to integrins α5β1 and αVβ3, but this ligand binds to the integrin headpiece, thereby activating integrin and the focal adhesion kinase pathway [103]. Caveolin-1, the major lipid raft marker, was also found in high concentrations in integrin α6β4 fractions, an interaction which contributed to tissue regeneration via the lipid raft-mediated integrin signaling pathway [104].

## 7. The Role of Cholesterol

Cholesterol is found in both leaflets of the membrane bilayer [105]. Its concentration directly influences integrins such as integrins αVβ3 and α5β1 [27,91] by affecting both adhesion and signaling by integrins. There is a strong correlation between membrane cholesterol and integrin signaling and adhesion, given that integrin assembly at cellular focal adhesions is dependent on the membrane cholesterol level [16]. While focal adhesions are highly enriched in cholesterol, cell detachment internalizes lipid rafts and decreases lipid order and the membrane cholesterol level. Further, a synthetic bile acid-phospholipid conjugate that inhibits integrin causes integrin internalization via lipid rafts [106]. Indeed, integrin β1 colocalizes with cholesterol in lipid rafts while ionizing radiation separates integrin β1 from cholesterol rafts [107]. Clearly, integrin adhesion and signaling correlates with the recruitment of these membrane proteins to lipid rafts. 

Integrin-mediated cell adhesion to the extracellular matrix regulates the localization of cholesterol-enriched vesicles to the membrane [93], resulting in altered membrane composition. The high concentration of cholesterol in focal adhesions increases the membrane order, and adhesion sites in general have a similarly high membrane order as seen in rafts [108] and a higher order compared to caveolae [108]. Cell detachment triggers the internalization of cholesterol accompanied by decreased lipid order and plasma membrane cholesterol concentrations, as well as the lipid raft marker, glycophosphatidylinositol-anchored protein [16]. Further, during heart disease and metabolic disorders, cholesterol increases in the tissue [109,110,111,112] and this likely is affecting integrin signaling through lipid raft regulation [111].

Integrin sequestration changes are also dependent on cholesterol concentrations in lipid rafts [113]. Ligands and bilayer asymmetry influence the sequestering of integrin into rafts and suggest a role of lipid packing and bilayer thickness that characterize the liquid-ordered and liquid-disordered domains in integrin sequestering. For example, integrin αVβ3 is sequestered into the liquid-disorder region in the absence of ligands but into the liquid-ordered domains upon binding to vitronectin. Despite the clear significance of cholesterol in integrin sequestering and recruitment during raft-mediated integrin adhesion and signaling, little is known about this biophysical regulation, probably due to the challenges attributed to the size and transient nature of raft domains in the plasma membrane [22,114]. 

Recent data showed that systematic changes in the membrane cholesterol concentration impact the sequestration of integrin αVβ3 in coexisting liquid-ordered and liquid-disordered domains, thus being qualitatively distinct in sequestering in the absence and presence of its native ligand vitronectin [113]. Nevertheless, the role of cholesterol in integrin sequestration and recruitment during lipid membrane raft-mediated integrin signaling and adhesions remains poorly understood, perhaps partly due to the small size and transient nature of raft domains [22,114].

## 8. Integrin-Glycosphingolipids Interactions

Mixtures of cholesterol, 1,2-dioleoyl-*sn*-glycero-3-phosphocholine, and dipalmitoylphosphatidylcholine form coexisting liquid-ordered and liquid-disordered domains, whereby molar concentrations influence integrin αVβ3 sequestration regardless of its ligand vitronectin and without clustering [113]. Significantly, focal adhesions have high concentrations of cholesterol [108] and can thus play a role in regulating integrin function [16]. Additionally, focal adhesions sense force and alter in size upon environmental stimuli during cell spreading and cell migration, whereby integrins diffuse into and out of focal adhesions [115,116,117].

Glycosphingolipids that are mainly in the outer leaflet also modulate integrin activity [118], whereby galacto-gluco-ceramide-mono-*N*-acetyl neuramic acid directly binds to integrin α5β1. Clustering of ganglioside-enriched lipid rafts regulate the activity of integrin β1 [119]. Further, integrin α5 was found to be the protein most elevated from lipid rafts of endothelial cells that was exposed to oscillatory shear stress, which also increased the level of activated integrin α5 regulated by membrane cholesterol and fluidity [120,121]. Lipid raft molecules dissociate upon cholesterol removal [122]. In vivo studies suggested a mechano-transduction mechanism that is integrin- and raft-dependent [120].

## 9. Role of Raft Thickness

In addition to native ligands, the bilayer asymmetry also influences the sequestration of integrins in raft-mimicking liquid mixtures [123,124]. The bilayer asymmetry dependency during integrin sequestration demonstrates the significance in the different lipid packing and bilayer thickness between liquid-ordered and liquid-disordered domains. As discussed above, cholesterol might regulate integrin distribution by changing the bilayer thickness and lipid packing densities in liquid-ordered and liquid-disordered domains. The cholesterol-mediated integrin sequestration is due to the bilayer thickness of coexisting liquid-ordered and liquid-disordered domains, whereby cholesterol might regulate the distribution of integrin αVβ3 by altering the bilayer thickness and lipid packing densities of the liquid-disordered and liquid-ordered domains [113]. 

The greater thickness of the ordered lipid structure in the raft domain compared to the non-raft membrane could be more favorable for the activated integrin transmembrane domain structure. Thereby, lipid rafts might stabilize the activated integrin conformation. In analogy with the immune system and the T cell receptor activating components of the signaling complex pre-assembled in the lipid rafts, these microdomains might provide a favorable environment in integrin clustering and maintaining downstream signaling [125]. 

The lipid bilayer separates its components laterally [6] and the domain formation is controlled by the bilayer thickness mismatch [126,127]. Cholesterol enhances lipid packing and bilayer thickness, whereby 29 mol% of cholesterol in a 1,2-dioleoyl-*sn*-glycero-3-phosphocholine bilayer increases its thickness by 2.8 Å [128]. This affects the hydrophobic matching conditions between the thicknesses of hydrophobic membranes versus the transmembrane region of membrane proteins. The regions of thick and thin membranes within the plasma membrane result from spontaneous partitioning of long-chain saturated lipids from short-chain polyunsaturated lipids into liquid-ordered and liquid-disordered domains [6,7]. While the reorganization of lipid phases and integrins plays a role in cell adhesion [6,71], it is unclear whether the liquid-ordered or liquid-disordered phases drive integrin reorganization. Integrin imaging by direct stochastic optical reconstruction microscopy (dSTORM) and stimulated emission depletion (STED) showed that the active and inactive forms of integrin β1 are localized to distinct types of clustering, but with which lipid domains the integrins were associated was not determined [129]. Since the membrane order is decreased by the detachment of integrin-mediated adhesion from a substrate [93,108], adhesion likely causes the transition from the liquid-ordered or liquid-disordered phase (Figure 3). In the absence of vitronectin, integrin αVβ3 shows a preference for liquid-disordered domains [113], which suggests a significance of domain-specific lipid packing as a biophysical regulator of integrin sequestration. Indeed, incorporation of membrane-spanning proteins into the cholesterol-enriched microdomains is energetically unfavorable [130] and not due to the hydrophobic mismatch, since the thickness of the integrin α and β transmembrane α-helices are 31.6 ± 3.4 and 30.0 ± 3.6 Å, respectively (https://opm.phar.umich.edu/), which is similar to the hydrophobic thickness of the liquid-disordered phase (33 ± 1 Å). However, upon binding to vitronectin, integrin αVβ3 translocates to the liquid-ordered domain, which is not due to the lipid packing differences, as here the hydrophobic mismatch is significant with the liquid-ordered thickness of 38 ± 1 Å [131] representing the biophysical mechanism of integrin sequestration. Thus, integrins might adjust their structure to overcome the hydrophobic mismatch as seen, for example, for rhodopsin, which adjusts its conformation or oligomerizes according to the bilayer thickness [132]. Interestingly, mutations of integrin αIIbβ3 that affect integrin activation [63] have perturbed transmembrane α-helix packing with crossing angles of the two integrin transmembrane α-helices 10° for the mutant, compared to 35° for the wild type [133].

Integrin α5β1 plays important physiological roles in metabolic syndrome and coronary heart disease. For example, inflammation activates β1 to cause contraction of endothelial cells and vascular leakage [134]. Inflammation also causes an influx of cholesterol, which potently alters lipid raft structures [111] and the thickness of the membrane. The transmembrane interactions of the α and β subunits keep integrin in its inactive, low affinity conformer [49,63,135,136]. In this inter-subunit interaction, integrin β is tilted by at least 25° [62,66,137,138] (Figure 3 and Figure 4). For integrin β, the thicker rafts might accommodate activated integrin by decreasing the integrin β transmembrane tilt [139]. However, this mechanism does not seem possible for integrin α, which is already almost completely straight in its inactive conformer [66,138]. Therefore, integrin α might instead overcome its hydrophobic mismatch in the thicker rafts by oligomerization (Figure 3 and Figure 4). Indeed, transmission electron microscopy of purified integrin αIIbβ3 showed clear dimers and trimer when incubated with activating manganese, which were not seen when incubated with calcium [140]. An integrin αIIb homodimer model confirmed this possibility [141] and a peptide of its transmembrane region oligomerizes in sodium dodecyl sulfate-polyacrylamide gel electrophoresis [142]. Integrin αα and ββ homodimers were found in zwitterionic or acidic micelles [143] as well as in biological membranes [141,143,144], which might function in integrin clustering by binding to multivalent ligands [49,145].

In general, the dimerization of transmembrane α-helices is energetically driven by the hydrophobic mismatch [146]. For example, the thickness of the lipid bilayer influences the monomer-dimer equilibrium of glycophorin A that dimerizes most efficiently under hydrophobic matching conditions [147]. That study further showed that cholesterol promoted self-association of transmembrane α-helices by affecting the order of the lipid acyl chains. Indeed, the lipid acyl chain order generally determines the strength and stability of transmembrane helix-helix interactions [140]. Alternatively, clustering in itself might drive the ordering of lipids and thus create a lipid raft, given that areas of higher lipid order would tend to fuse and thereby favoring the clustering of signaling molecules [83]. While the role of anionic lipids in inside-out signaling is starting to be appreciated, the molecular mechanisms remain controversial [148,149,150].

## 10. Phosphatidylinositol 4,5-Bisphosphate (PI(4,5)P_2_) Lipid Microdomains

Another form of lipid organization occurs with negatively charged phospholipids, such as phosphatidylinositol 4,5-bisphosphate (PI(4,5)P_2_), which plays key roles in cell adhesion. PI(4,5)P_2_ is present in the inner leaflet of the plasma membrane at relatively low concentrations overall (~1% in average), but is enriched at focal adhesion sites by the enzyme phosphatidylinositol 4-phosphate 5-kinase type Iγ (PIP5KIγ) [151,152]. The longer PIP5KIγ668 isoform (human numbering) contains additional *C*-terminal residues that target the enzyme to focal adhesions by interacting with the talin head domain [153,154]. In the presence of bivalent cations or basic peptides, PI(4,5)P_2_ can cluster into microdomains with a very high PI(4,5)P_2_ content [155,156]. Formation of PI(4,5)P_2_ microdomains was demonstrated in model membrane systems in the presence of calcium and to a lesser extent with magnesium [155,157], but has also been observed in cells [158,159]. Using super-resolution stimulated emission depletion microscopy, PI(4,5)P_2_ microdomains were observed in pheochromocytoma (PC) 12 cells associated with the SNAP receptor protein syntaxin-1A and were found to have a size of approximately 70 nm and an estimated PI(4,5)P_2_ content as high as 82% [159].

How these PI(4,5)P_2_ microdomains interact or coexist with cholesterol-rich lipid rafts has been a matter of debate. Initial studies suggested that PI(4,5)P_2_ partitions into lipid rafts based on the fact that depletion of cholesterol lead to delocalization of PI(4,5)P_2_ microdomains [160]. This view was supported by the fact that several signaling events are apparently triggered by both lipid rafts and associated PI(4,5)P_2_ microdomains [161,162]. However, other studies have refuted the existence of PI(4,5)P_2_ inside lipid rafts [163], arguing that the unsaturated acyl chains found in PI(4,5)P_2_ would indeed not seem compatible with an ordered lipid raft domain. Nevertheless, multiple studies show that cholesterol promotes PI(4,5)P_2_ clustering into microdomains [164,165,166]. The exact mechanism for this is unclear, but altering the charge state of PI(4,5)P_2_, providing hydrogen bonds to the PI(4,5)P_2_ headgroup, or altering the lateral mobility of PI(4,5)P_2_ have been suggested as possible reasons [164,165,167].

Several of the core focal adhesion proteins interact with PI(4,5)P_2_, including talin, vinculin, and focal adhesion kinase [148,168,169,170,171,172,173]. In all these cases, PI(4,5)P_2_ binding induces conformational changes, which result in enhanced adhesion strength by talin and vinculin [149,174] or increased adhesion signaling via focal adhesion kinase [175,176]. As discussed above for lipid rafts, an intriguing question is whether PI(4,5)P_2_ microdomains may play a role in integrin clustering and/or assembly and stabilization of the mature focal adhesion complex. This might occur either via colocalizing focal adhesion proteins to PI(4,5)P_2_ membrane domains due to the high local density of PI(4,5)P_2_, or by inducing more stable protein assemblies, such as oligomeric states of focal adhesion proteins. Interestingly, PI(4,5)P_2_ is found to induce oligomerization and clustering in various systems also outside focal adhesions, including the adhesion molecule CD44 or the serotonin transporter SERT [177,178]. In focal adhesions, PI(4,5)P_2_ is shown to promote oligomerization of vinculin and focal adhesion kinase. In the case of vinculin, PI(4,5)P_2_ bridges vinculin molecules via different binding sites to promote higher-order oligomers [170,171,173], whereas for focal adhesion kinase, binding to PI(4,5)P_2_ induces conformational changes that promote focal adhesion kinase oligomers via protein/protein interactions [175]. Whether such effects can contribute to integrin clustering is unclear, but based on observations discussed above, the presence of high density PI(4,5)P_2_ microdomains can be expected to have important roles in adhesion stability, maturation, and signaling via a number of mechanisms, such as promoting colocalization, oligomerization, and conformational changes in a number of focal adhesion proteins. 

## 11. The Role of Integrin Acylation

In general, acylation (addition of myristol or palmitoyl) but not prenylation (addition of farnesyl or geranyl-geranyl) targets proteins to lipid rafts [179,180]. Myristoyl or prenyl group attachment mainly results in higher affinity for membranes and promotes intramolecular and intermolecular protein-protein interactions [181]. While geranylgeranyl can anchor proteins in the membrane, farnesyl cannot [181]. For example, integrin β4 is palmitoylated on several cysteines (residues 732, 736, 738, 742) at the membrane-proximal segment of the β4 tail [84,182,183,184], residues that are not conserved in platelet integrin β3 [62], which is important for membrane binding and lipid raft localization [181] and is necessary for integrin α6β4 incorporation into lipid rafts where it interacts with growth factor receptors and enhances invasiveness of cancer cells [84,185,186]. Further, palmitoylation of integrins α3, β4, and α6 affects their interaction with tetraspanin [185,187].

Integrins α3β1 and α6β1 are not palmitoylated [84] and might associate with tetraspanins [188] which are palmitoylated and could promote integrin incorporation into lipid rafts [84]. The concept that a palmitoylated protein moves between a ganglioside (GM1) raft and PI(4,5)P_2_ domains was first described by Hansen [189]. While palmitoylation of integrin β4 is necessary for integrin α6β4 incorporation into lipid rafts, it is not required for its binding to laminin-5 nor assembly of hemidesmosomes or adhesion [84]. This again suggests that the membrane plays a role in integrin activation. More studies are required to better understand the role and mechanism of acylation of integrins during integrin activation.

## 12. Conclusions and Future Directions

Lipid rafts regulate signaling pathways such as integrin signaling by partitioning activated integrins in these microdomains where they form specific interactions with upstream and downstream signaling molecules. Lipid rafts might sequester activated integrins by providing a more favorable membrane environment for the distinct conformation of activated integrins. Two decades ago, tilting of the transmembrane domain of inactive integrins was predicted and then confirmed experimentally [61]. At the same time, a slight movement of one or both integrin transmembrane domains in and out of the membrane with interacting proteins to mask exposed integrin transmembrane regions was already noted as a mechanism for integrin activation [65,190]. However, despite decades of studies on integrin activation, the roles that the lipids in the plasma membrane play in these inside-out and outside-in activation steps are still not fully understood. The complex lipid/protein environment in lipid rafts is challenging to reconstitute in vitro, hence no high-resolution structural information is currently available that could provide detailed mechanistic insight into lipid-mediated integrin activation. Future advances that will help to better understand the lipid raft structure and what exactly stabilizes them together with the recent revolution in cryogenic electron microscopy are likely to provide such insights at atomic detail. Such studies will be directly relevant to our understanding of complex diseases in humans.
